# Nanocomposites for Electrochemical Sensors and Their Applications on the Detection of Trace Metals in Environmental Water Samples

**DOI:** 10.3390/s21010131

**Published:** 2020-12-28

**Authors:** Tshimangadzo S. Munonde, Philiswa N. Nomngongo

**Affiliations:** 1 Department of Chemical Sciences, University of Johannesburg, Doornfontein Campus, P.O. Box 17011, Doornfontein 2028, South Africa; 215077511@student.uj.ac.za; 2 DST/NRF SARChI Chair, Nanotechnology for Water, University of Johannesburg, Doornfontein 2028, South Africa; 3 DST/Mintek Nanotechnology Innovation Centre, University of Johannesburg, Doornfontein 2028, South Africa

**Keywords:** nanomaterials, nanosensors, sensitivity, selectivity, trace metals, nanocomposites

## Abstract

The elevated concentrations of various trace metals beyond existing guideline recommendations in water bodies have promoted research on the development of various electrochemical nanosensors for the trace metals’ early detection. Inspired by the exciting physical and chemical properties of nanomaterials, advanced functional nanocomposites with improved sensitivity, sensitivity and stability, amongst other performance parameters, have been synthesized, characterized, and applied on the detection of various trace metals in water matrices. Nanocomposites have been perceived as a solution to address a critical challenge of distinct nanomaterials that are limited by agglomerations, structure stacking leading to aggregations, low conductivity, and limited porous structure for electrolyte access, amongst others. In the past few years, much effort has been dedicated to the development of various nanocomposites such as; electrochemical nanosensors for the detection of trace metals in water matrices. Herein, the recent progress on the development of nanocomposites classified according to their structure as carbon nanocomposites, metallic nanocomposites, and metal oxide/hydroxide nanocomposites is summarized, alongside their application as electrochemical nanosensors for trace metals detection in water matrices. Some perspectives on the development of smart electrochemical nanosensors are also introduced.

## 1. Introduction

The key factor to mitigating trace metals pollution and keeping the water quality up to standard is the early detection of trace metals within the environmental water bodies [1]. Undoubtedly, the global economic expansion and industrial developments necessary to meet demand and the continued sustainability of human lives have been appreciated worldwide. At the same time, the discharges from the increased chemical processes, agricultural processes, and energy conversions, amongst other applications, have elevated water pollution. These anthropogenic processes have been observed to be the major contributors to the elevated concentrations of trace metals in water bodies [2,3,4,5,6]. The statistics published by the World Health Organization (WHO), based on the 2017 figures, pronounce that 785 million people lack basic drinking-water services, including 144 million people who are dependent on surface water. Globally, at least 2 billion people use a drinking water source contaminated with faeces, leading to the transmission of diseases such as; diarrhea, cholera, dysentery, typhoid, and polio, amongst others. It is well known that a class of essential trace elements, such as; zinc (Zn), copper (Cu), molybdenum (Mo), selenium (Se), chromium III (Cr), cobalt (Co), manganese (Mn), amongst others, play significant roles in nutrition balance and other biological aspects [7,8]. However, their excessive ingestion above the maximum permitted guidelines provided by the World Health Organization (WHO) may lead to various adverse effects ranging from acute to chronic impacts, depending on the type, species and level of exposure [9,10]. Another class of toxic trace metals, comprised of mercury (Hg), arsenic (As), thallium (TI), tin (Sn), antimony (Sb) and lead (Pb), amongst others, are poisonous and provide minimal health benefits even at trace levels [7,11]. To avoid a trace metal-based catastrophe, the amount of trace metals, predominantly toxic trace metals, in environmental water bodies ought to be frequently monitored and regulated. Traditional methods for the detection and monitoring of trace metals in water bodies include, but are not limited to, spectrometric techniques such as; inductively coupled plasma-optical emission spectrometry (ICP-OES) [6], inductively coupled plasma-mass spectrometry (ICP-MS) [12], flame atomic absorption spectroscopy (FAAS) [13], graphite furnace atomic absorption spectroscopy (GFAAS) [14] and x-ray fluorescence (XRF) [15]. However, these techniques require sample collection, sample pre-treatment, high energy input, pure gas input, infrastructure and skilled engineers, scientists, or technicians to operate the instruments [6,12,13,14,15,16]. As an alternative to these techniques, electroanalytical devices, particularly electrochemical sensors, have been identified as potential candidates for the low cost, sensitive and selective detection of trace metals in environmental water bodies [17,18,19]. Nowadays, nanomaterial technology plays an important role in providing opportunities and possibilities for the development of a new generation of sensing tools [20,21]. Considering their low cost, high efficiency, multiple functionality, flexibility, selectivity, and sensitivity, nanosensor devices account for the shortcomings encountered by the traditional analytical detection techniques [22]. Due to the incorporation of nanomaterials into their working electrode systems, significant enhancements on the sensitivity and selectivity of electrochemical nanosensors, amongst other performance parameters, have been reported [23]. It is without any doubt that the properties of nanomaterials, such as; the shape, size, aggregation/agglomeration state, size distribution, crystallinity and defect structure, play a significant role on the electrode’s performance in electrochemical sensors [24,25,26]. Tuning/engineering the nanomaterials on the surface of the electrode enables the detection of various toxic trace metals simultaneously or independently [27,28]. In the past few decades, extensive research has been carried out on electrochemical sensors incorporating engineered carbon [29,30], transition metals [31,32], and transition metal oxide/hydroxide [33,34] nanostructured materials as recognition elements, with promising results reported. To this end, three major strategies, including the engineering of active sites, engineering the electronic conductivity, and constructing a porous structure in nanomaterials, have been identified to improve the electrode’s surface chemistry and therefore enhance the electrode’s performance [35,36,37,38]. On the basis of this rationalization, we systematically reviewed the recent nanomaterials, specifically nanocomposites, for electrochemical nanosensors and their applications in toxic trace metal detection in environmental water samples. Current developments have also seen attempts to use smart technology, incorporating 4IR technologies in nanosensors to produce smart nanosensor devices for monitoring trace metals within the environment. Following the comprehensive review of the nanosensors and their applications, smart nanosensors were evaluated and key concerns, challenges, and future forecasts in the monitoring and detections of trace metals in environmental water bodies were deliberated.

## 2. Nanosensors

**Scheme 1:**
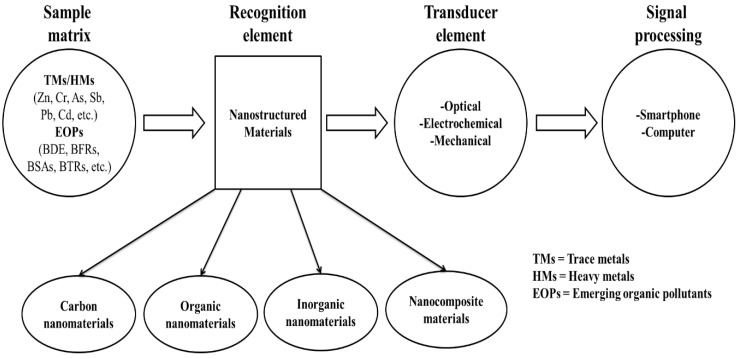
Operational mechanisms of nanosensors.

Nanosensors are sensing devices that are dependent on the unique properties of nanomaterials to recognize and detect the behavior of matter within a given environment at the nanoscale to macromolecular level [22]. These sensing devices can be large or small devices that utilize the nanomaterials incorporated as recognition elements to detect changes at a nanoscale [39]. The sensing mechanism follows either the optical, electrochemical or mechanical route of detection [22,40], as a result of the combination of the recognition element and the detector used as illustrated in Scheme 1. Evidently, as key technological and economic drivers, various nanosensors have been developed for the detection of chemical and toxic gases, food packaging, medical diagnostics, and water quality monitoring, amongst other applications [41,42,43]. Interestingly, the recognition element has been identified amongst these applications as the key element giving the identity of the nanosensor [44]. Owing to their unique physico-chemical properties enhancing the signal detection, nanomaterials have been incorporated as recognition elements in sensors [22]. Nanomaterials allow for the amplified binding of target molecules on their available active sites, which lead to detectable signals [44]. It is worth noting that if the recognition element is a nanostructured material, then a nanosensor is obtained [22]. To this end, various classes of nanostructured materials ranging from carbon-based nanomaterials to organic-based nanomaterials, inorganic-based nanomaterials, and nanocomposite materials (incorporated in Scheme 1) have been synthesized and deployed in the afore-mentioned applications [41,42,43]. Various synthetic methods for the synthesis of these classes of nanomaterials are outlined in the next section.

## 3. Nanosensor Fabrication

Nanomaterials properties such as; high surface area-volume ratio, dimensionality (<100 nm), high adsorption surface area, high mobility of particles, reproducibility of the particles, uniformity, composition, rapid/delayed particle agglomeration and high electric/heat conductivity are crucial in nanosensors [45,46]. Miniaturization of the nanomaterials improves the surface-to-volume ratio of nanomaterials, resulting in variations in chemical, mechanical, optical, and magnetic properties, resulting in the increased sensitivity and specificity in nanosensors [47,48]. The nanomaterials synthesis method is therefore crucial for improving the performance of the nanosensors. Moreover, synthesis methods leading to the production of uniformly distributed particles and consistent shapes are necessary to improve the linearity and the detection limits of the nanosensor, amongst other performance parameters [47]. There are two common approaches for the synthesis of nanomaterials, namely top down and bottom up methods [49]. Common top down methods involve mechanical methods such as; mechanical grinding, high energy ball milling, mechanical alloying, reactive milling and lithography [16]. Common bottom up methods include chemical methods such as; hydrothermal, co-precipitation, microemulsion, sol-gel, chemical-vapor deposition, electrodeposition, epitaxial growth, and colloidal dispersion [50]. 

From the perspective of this review, the classification of the nanomaterials was done based on the structural composition, such as; carbon based nanomaterials, metallic nanomaterials, and metal oxides/hydroxide nanomaterials. After the identification and description of the key performance parameter, the three classes’ classifications are used in Section 5 to discuss various nanomaterials for sensing trace metals in environmental water samples for water quality assessment applications.

## 4. Performance Parameters of Nanosensors

**Figure 1:**
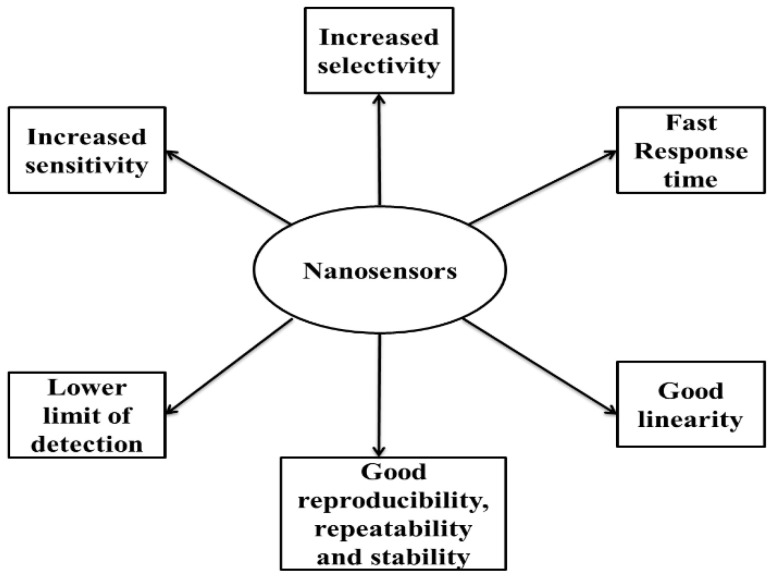
Key performance parameters of nanosensors.

Since the first report on the use of nanomaterials in sensors to improve their performance, extended efforts have been made to synthesize various nanomaterials and applying them as nanosensors for the detection and monitoring trace metals in water [51,52,53]. To this effect, a variety of parameters influencing the extended improvements to nanosensors have been assessed. These include, but are not limited to, the increased sensitivity, increased selectivity, lower detection limit, fast response time, good linearity, stability and the extended lifetime [47,54,55,56,57], as shown in Figure 1. 

### 4.1. Increased Sensitivity

The sensitivity of a sensor is generally defined as the ratio between a physical parameter and the output signal generated from that physical parameter [58]. The general equation (Equation (1)) is shown below:

Sensitivity (S) = ΔY/ΔX(1)

where Y = output signal variation and X = input signal variation.

It has been noted that improving the conductivity of the sensor increases the sensitivity of the sensor. Through their increased surface area to volume ratio, nanomaterial-based sensors have been shown to possess an increased sensitivity over traditional sensors [59]. The increased sensitivity is often ascribed to the improved conductivity resulting from the controlled dimensions and shapes leading to increased electrical and optical properties, in electrochemical and optical nanosensors, respectively [22,60]. Furthermore, it has been reported that any type of anisotropy in the shape of the nanomaterial, including the aggregation of the nanomaterial, can greatly enhance the signal amplification and thereby enhance the sensitivity of the nanosensor [61]. 

### 4.2. Good Linearity

The linearity is related to the operational window of the sensor discriminating the actual measured curve from the model curve. This is formulated as a range from the minimum to maximum permitted input and output estimates [62]. The linear relationship expressed in Equation (2) is a key fundamental equation that needs to be satisfied by any sensor’s linear calibration curve.

Y = mX + c(2)

where Y = output signal, X = input signal, m = slope/sensitivity of the sensor, and c = the constant parameter related to the baseline offset.

In addition to the above linear relationship, the correlation coefficient of the calibration curve is deemed critical in evaluating the linearity of the designed sensor. However, according to the FDA, statistical methods such as; ANOVA or standard relative deviation in particular can be used to evaluate the linearity of the calibration curve(s) [39,62]. Subsequently, nanomaterial-based sensors have been reported to show a good linearity as a result of the reproducibility and uniformity of the nanomaterials adopted on the generation of the calibration curves [39,63].

### 4.3. Lower Limit of Detection

The limit of detection (LOD), defined as the lowest amount of the analyte in the test sample that can be reliably distinguished from zero, is a key parameter in sensors that intertwines both the sensitivity and the theoretical instrument resolution [64,65]. The universal expression used in the determination of the LOD is shown in Equation (3).

LOD = 3(SD/S)(3)

where SD = standard deviation of the blank solution and S = slope of the calibration curve.

Traditional sensors suffer greatly from the generation of non-linear calibration curves, leading to the over/under assessment of the target analyte(s) [56]. Nanomaterials have provided a feasible solution owing to the reproducibility of the particles and the uniformity in size and shape. These significant properties of nanomaterials enable the linear calibration curves generation leading to the increased sensitivity of the target analyte(s). Furthermore, the increase in the surface area resulting in the miniaturization of materials has shown to result in low LODs [60]. The signal-noise ratio is also reduced with the application of nanomaterials in sensors [57].

### 4.4. Increased Selectivity

Selectivity refers to the capability of the sensor to simultaneously discriminate the response of other competing substances whilst generating the maximum output of the target analyte(s) [66]. The low selectivity is a major drawback for sensor deployment in environmental-based applications. However, the emancipation of nanomaterial-based sensors has successfully reformed these dynamics. Nanomaterials with unique shapes corresponding to the target analyte have been prepared to selectively target the analyte of interest from competing substances within the complex sample matrix [26,66]. Additionally, the increase in the binding sites resulting from the increase in the surface to volume ratio of the nanomaterials has been shown to increase the selectivity of the nanosensors [60]. Thus, tuning the nanomaterial structure plays a significant role in the selectivity of the nanosensors.

### 4.5. Fast Response Time

The responsiveness of any sensor is usually defined according to the time (or frequency in some cases) taken to effect an applied input parameter change [67]. Evidently, the input parameter change in sensors does not come into effect immediately; instead, it takes a certain period of time, known as the response time [60]. Interestingly, the response times of the nanosensors have been observed to be lower than those of traditional sensors [68]. This denotes that the nanomaterial-enabled sensors respond quicker to the input parameter change than the traditional sensors. 

### 4.6. Good Reproducibility, Repeatability and Stability

Sensors reproducibility and repeatability, represented as an inter-day and intra-day precision, is one of the most important parameters to consider whilst assessing the performance of a sensor [69]. Equally, the ability of the sensor to withstand long operational hours under hostile environments is monitored [70]. Nanosensors often retain their compact structure even after operating repeatedly for a long time period without a significant decrease in the performance, as opposed to traditional sensors that appear to degrade in performance when used for a long time under hostile environments [71]. 

Now that the key performance parameters are known and assessed, various materials are reviewed against these parameters following the three afore-identified classes of nanomaterials. 

## 5. Nanocomposites in Electrochemical Sensors for Trace Metal Detection

### 5.1. Carbon Based Nanomaterials

Compared to their bulky counterpart, carbon-based nanomaterials possess extraordinary properties, such as; high surface area, electrical conductivity, heat conductivity, and mechanical properties [72]. As a result, various carbon nanomaterials and their derivatives, such as; graphene, carbon nanotubes (CNTs), reduced graphene oxide (rGO), graphitic carbon nitride (g-C_3_N_4_) and carbon nanofibers (CNF), have been adopted for use in electrochemical nanosensors for trace metal detection in water. To this end, the synthesis, post-treatment, optimization and characterization of the carbon-based nanomaterials, and the influence of their structural properties in electrochemical sensors, have been explored. Although various carbon nanomaterials have been synthesized, graphene derivatives (rGO and graphene oxide (GO)) and g-C_3_N_4_ have been shown to be the most ideal candidates for electrochemical sensors as a result of their extraordinary physical and chemical properties. For the purpose of this review, we will focus on the discussions of graphene derivatives (rGO and GO) and g-C_3_N_4_-based materials, with some examples of other carbon materials shown in Table 1. 

#### 5.1.1. Graphene-Based Nanocomposites

**Figure 2:**
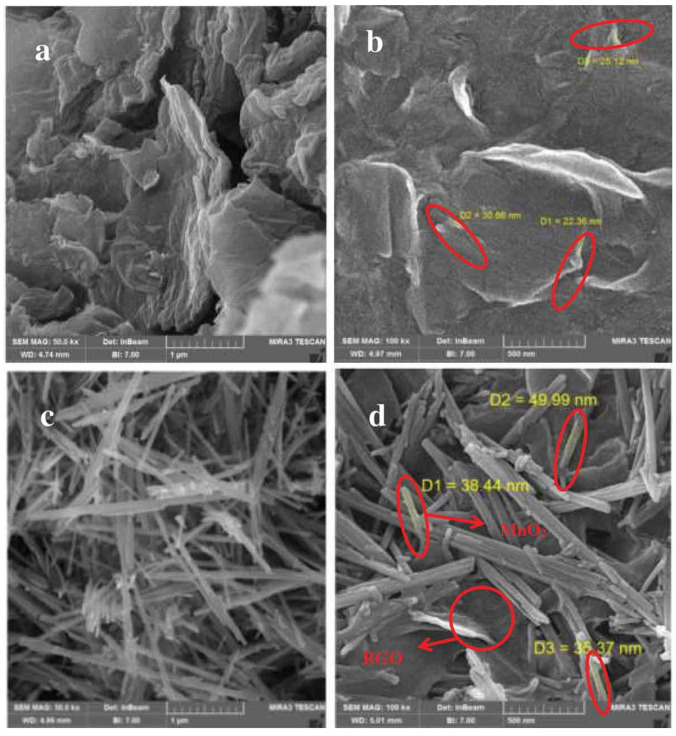
FE-SEM images of (**a**) graphene oxide (GO), (**b**) reduced graphene oxide (rGO), (**c**) α-MnO_2_, (**d**) α-MnO_2_/rGO nanocomposite (reproduced with permission from [81]; copyright 2019, Taylor & Francis Group).

Graphene, GO, and rGO form part of the graphene nanostructured family consisting of 2D carbon atoms arranged in a single-layer hexagonal honeycomb pattern [73]. Owing to the high electrical conductivity, large surface area, chemical stability, environmental friendliness and high electrocatalytic activities of graphene, graphene-based nanostructures have attracted extensive interest in electrochemical applications [74]. However, considering the hydrophobic nature of graphene and the inability to form stable homogenous dispersions, the highly oxidized GO and the less oxidized rGO have played a key role in electrochemical reactions [73,75,76]. Subsequently, GO possesses oxygenated graphene sheets with hydroxyl and epoxy groups on the basal plane alongside carboxyl and carbonyl groups on the edges, leading to an intensive interaction with a variety of metal ions [74]. However, the high concentration of sp^3^ C-O bonds in GO decreases the electron transfer, which therefore reduces the electron conductivity [77]. To this end, the reduction of GO via the removal of some oxygen based groups by chemical/thermal treatment forms rGO with increased electron conductivity [78]. Although the reduction of GO to rGO leads to abundant structural defects (oxygen vacancies) and desired functional groups influencing their use in electrochemical sensors, the aggregation and π−π stacking interactions of graphene layers still persists [79]. To overcome these drawbacks, various materials including; metal nanoparticles, conducting polymer, and silver nanowires have been incorporated into rGO suspensions [77,79]. Engineering the electronic conductivity by the introduction of oxygen vacancies (through the reduction of GO to rGO) [77,78] and the doping of the rGO materials with other heteroatoms appears to be an effective strategy to improve the rGO electrochemical activity [80]. To attempt this, Hassanpoor and Rouhi [81] incorporated the hydrothermally prepared MnO_2_ nanorods on rGO layers using the facile mixing and stirring method for the determination of trace amounts of cadmium (II) in environmental water samples. Post the reduction of GO to rGO, the restacking and aggregation of the rGO layers were observed on the SEM images, which necessitated the incorporation of MnO_2_ nanorods (Figure 2). The close contact between rGO nanosheets and MnO_2_ nanorods was also observed on the SEM images, which has proven to be very important for improving the high degree of electrical conductivity. Under optimum conditions, the α-MnO_2_/rGO nanocomposite displayed good analytical figures of merits with a linear range and LOD of 4.0 to 130 μg L^−1^ and 1.12 μg L^−1^ for Cd (II), respectively. The application of the α-MnO_2_/rGO nanocomposite in tap water and river water displayed good recoveries of 103.8 and 102.4%, respectively, which were attributed to the synergic effect of rGO resulting on the good conductivity and high surface area, as well as the good catalytic activity of MnO_2_ nanorods that can form other species of manganese such as; MnOOH, Mn^2+^, Mn(OH)_2_, Mn_3_O_4_ in solution. The electrochemical sensor also demonstrated higher sensitivity and selectivity towards the detection of Cd^2+^ in a complex matrix with other co-existing metal ions. Although this strategy yielded good results, it appears less feasible than the doping with polymers [30] and binder specific aptamers [40]. Further examples of the rGO-based nanomaterials as electrochemical sensors for a variety of trace metals are shown in Table 1.

#### 5.1.2. Graphitic Carbon Nitride-Based Nanocomposites

**Scheme 2:**
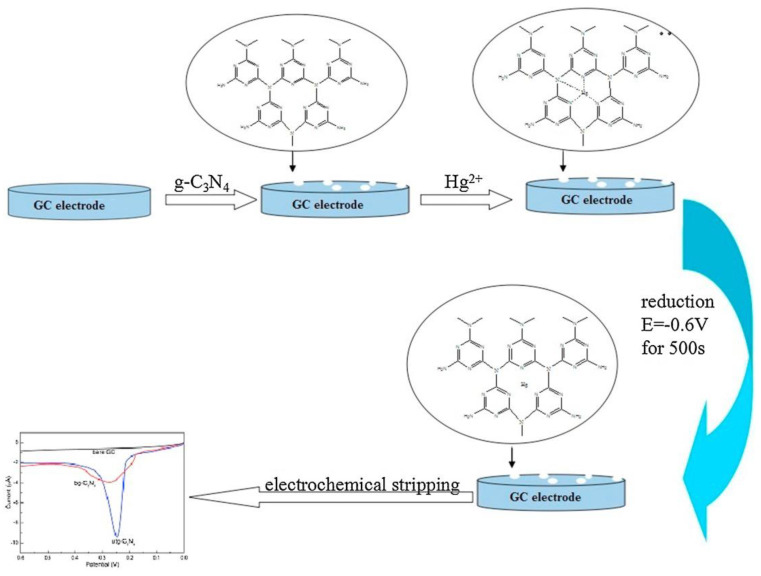
Illustration of the electrochemical sensing Hg^2+^ ions using ultrathin g-C_3_N_4_/GCE electrode (reproduced with permission from [87], copyright 2015 Elsevier Ltd.).

As a derivative of graphite exhibiting a unique electronic structure, graphitic carbon nitride (g-C_3_N_4_) has attracted significant attention in various electrochemical applications, such as; water splitting, photocatalysis, sensors, batteries, and fuel cells, amongst others [82,83]. However, the widespread applications of g-C_3_N_4_ in electrochemical sensors have been limited by the low electrical conductivity and a small specific surface area [84]. However, inspired by the presence of N and H atoms forming an electron-rich graphitic like tri-s-triazine units, whose layers are combined together with weak van der Waals forces, g-C_3_N_4_ has been regarded as a potential candidate to complement carbon in various material-based applications. The interlayer distance between g-C_3_N_4_ layers allows for the ultrasonic exfoliation into g-C_3_N_4_ nanosheets [85] or g-C_3_N_4_ nanorods [86], leading to more engineered active sites due to an increase in the surface area. To demonstrate this, Zhang et al. [87] exfoliated the bulky g-C_3_N_4_ prepared by thermal polycondensation to produce ultrathin g-C_3_N_4_ nanosheets employed as a sensing platform for the determination of trace mercury (Hg^2+^) using an anodic stripping voltammetry (ASV) technique in water samples (Scheme 2). For this purpose, the ultrathin g-C_3_N_4_ nanosheets modified glassy carbon electrode (GCE) with increased surface sites showed enhanced electrochemical response to Hg^2+^ than the bulky g-C_3_N_4_ modified GCE. The strong affinity of Hg^2+^ and ultrathin g-C_3_N_4_ through surface -NH and -NH_2_ groups, leading to increased electron conductivity based on the lone pairs of electrons in N atoms, resulted in the high sensitivity and selectivity of the electrochemical sensor towards Hg^2+^ detection. Thus, under optimized conditions, the linear sweep anodic stripping voltammetry (LSASV) showed a linear response in the range of 0.1–15 ppm and a LOD of 0.023 ppm. The feasibility of the developed electrochemical sensing strategy was demonstrated by applying it for the detection of Hg^2+^ in various real samples including tap, lake, and river water, with acceptable recoveries ranging from 95% to 107.5% for the ultrathin g-C_3_N_4_/GC electrode, which agreed well with the ICP-AES measurements. 

**Figure 3:**
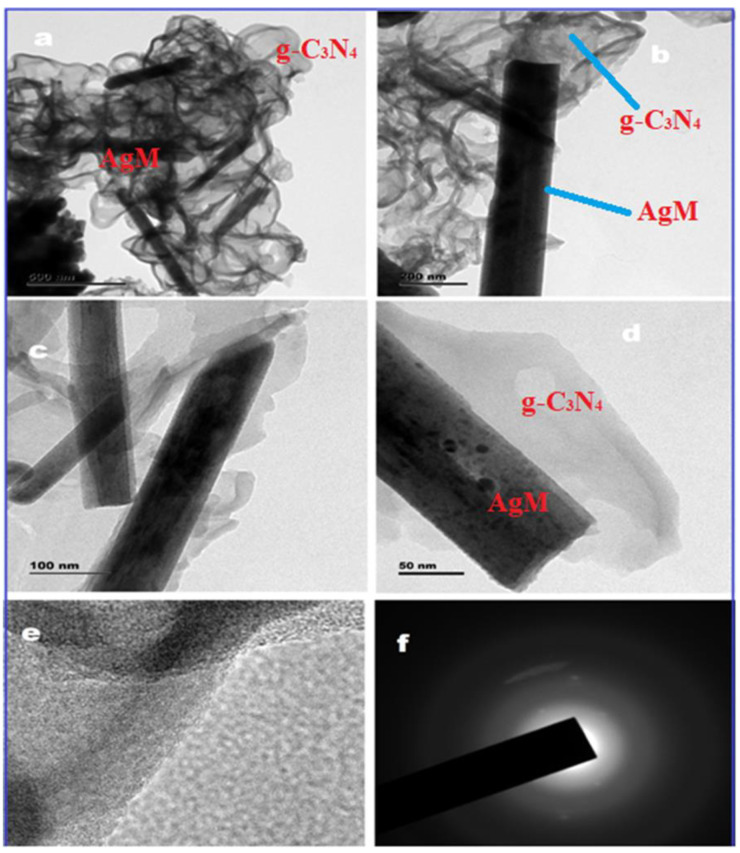
High resolution-transmission electron microscopy (HR-TEM)showing different magnification images of g-C_3_N_4_/AgM nanocomposites (**a**–**e**) and selected area electron diffraction (SAED) pattern of g-C_3_N_4_/AgM nanocomposite (**f**) (reproduced with permission from [89], copyright 2020 Elsevier B.V.).

Another strategy involves the surface modifications to further enhance the electronic conductivity by the incorporation of other conductive materials [84] or doping with other heteroatoms [88], leading to enhanced electrochemical properties of the g-C_3_N_4_ materials. To illustrate this, Karthika et al. [89] developed a graphene carbon nitride decorated with silver molybdate nanocomposite using a solvothermal method for the electrochemical detection of chromium (VI) in water samples. The TEM images (Figure 3) clearly show the interaction of g-C_3_N_4_ with AgM that resulted in the improvement of the electrochemical activity of the sensor. The amperometric electrochemical method showed tremendous sensitivity of 65.8 µA µM^−1^ cm^−2^, good linear ranges from 0.1 to 0.7 µM, and a lower LOD of 0.0016 µM that can be attributed to the availability of greater surface area and adsorption sites. Furthermore, the authors indicated that graphene carbon nitride-based metal oxides enhance the electrochemical sensing due to their large surface area, electrical conductivity, thermal conductivity, interlayer structure and stability. The electrochemical sensor was employed for the determination of Cr^6+^ in tap, drinking, river, and industrial water samples, respectively, with recoveries ranging from 94.3 to 100.2%. Additionally, the g-C_3_N_4_/AgM modified electrode exhibited good selectivity, stability, reproducibility and repeatability. Further examples on the g-C_3_N_4_-based nanomaterials for the electrochemical sensing of trace metals in water are shown in Table 1.

**Table 1:** Electroanalytical performances of selected carbon nanocomposite-based sensors for trace metal detection in environmental water matrices.

Nanomaterial/Electrode	Synthesis Method	Target Analyte	Technique	LOD	Linear Range	RSD (%)	n	Sensitivity	Ref
**g-C_3_N_4_/AgM/Nf/GCE**	Sonochemical	Cr^6+^	Amp	0.002 (µM)	0.1–0.7 (µM)	2.83	30	65.8 (µA µM^−1^ cm^−2^)	[89]
**Pt/g-C_3_N_4_/polythiophene/GCE**	Chemical reduction	Hg^2+^	DPV	0.009 (nM)	1–500 (nM)	1.80	3	1.08 (µA nM^−1^ cm^−2^)	[90]
**rGO/Ala/PANI/GCE**	Chemical reduction/polymerization	Cd^2+^ Pb^2+^ Cu^2+^	SWASV	0.030 (nM) 0.045 (nM) 0.063 (nM)	0.08–100 (nM)	3.30 2.60 3.00	10	0.43 0.71 0.61 (μA nM^−1^ cm^−2^)	[91]
**Nf/CLS/PGR/GCE**	Hydrothermal/thermal reduction	Cd^2+^ Pb^2+^	DPASV	0.010 (μM) 0.003 (µM)	0.05–5.00 (µM)	4.54 3.63	8	9.77 32.7 (µA µM^−1^ cm^−2^)	[92]
**Bi-NCNF/GCE**	pyrolysis	Cd^2+^ Pb^2+^	SWASV	0.020 (μM) 0.030 (μM)	1–120 (μM)	7.10 4.30	10	0.207 0.273 (μA µM^−1^)	[93]

n = reproducibility, GCE = glassy carbon electrode, Nf = nafion, PANI = polyanaline, CLS = calcium lignosulphonate, PGR = porous graphene, NCNF = N-doped carbon nanofibers, Amp = amperometry.

Table 1 shows different carbon-based sensors modified with various materials of different classes, ranging from non-noble metal nanoparticles (NPs) (Bi and AgM) to noble metal NPs (Pt), organic compounds (calcium lignosulphonate, CLS) and polymers with amino acids (polyaniline (PANI)/alanine), alongside their corresponding analytical responses. The modification of g-C_3_N_4_ with Pt and PTh in the Pt/g-C_3_N_4_/polythiophene/GCE nanocomposite for the detection of Hg^2+^ was observed to improve the LOD and sensitivity at a low % relative standard deviation (RSD), as shown in Table 1. In addition to the increased active sites, the improved performance was attributed to the presence of PTh and Pt NPs that compensated for the lower electrical conductivity of the g-C_3_N_4_ in the nanocomposite. The sensor also showed higher reproducibility. Likewise, the g-C_3_N_4_/AgM nanorods in the g-C_3_N_4_/AgM/Nf/GCE nanocomposite exhibited a large surface area and the electron transfer constant rate was enhanced by the grafted immobilized nafion. This resulted not only in the improved LOD, sensitivity and a low % RSD shown in Table 1, but also exhibited a good selectivity, stability, sensitivity, and reproducibility, which certified that the g-C_3_N_4_/AgM/Nf/GCE is a promising sensor. The rGO/Ala/PANI/GCE nanocomposite that exhibited high sensitivity and selectivity towards Cd^2+^, Pb^2+^, and Cu^2+^ ions detection which were attributed to the strong binding affinity of rGO/Ala/PANI nanocomposite with these target metal ions was proposed. Subsequently, the sensor displayed a wide linear range and low LODs (Table 1) which are accredited to the high surface binding affinity, improved electrical conduction path, electron tunneling, and ion-trapping properties, together with remarkable electrocatalytic activity. The Nf/CLS/PGR/GCE nanocomposite developed for the simultaneous detection of Pb^2+^ and Cd^2+^ showed low LODs at a wide linear range (Table 1), coupled with good sensitivity, repeatability and reproducibility. The improved performance could be ascribed to the synergistic contribution from porous graphene (PGR), CLS and nafion, which integrated the advantageous features of high active surface area, good cation exchange ability and strong adsorption capacity. The designed 3D Bi-NCNF/GCE nanocomposite was found to possess porous network structures, high specific surface area, and high nitrogen content dopant, and acts as an electrode-modification material for selective and sensitive electrochemical sensors for the assay of Cd^2+^ and Pb^2+^. Due to this, the sensor exhibited excellent electroanalytical performance with a wide linear response range, low LOD and low % RSD (Table 1), in addition to the excellent repeatability, reproducibility, and stability.

### 5.2. Inorganic Metallic Nanoparticles

Owing to their attractive physico-chemical properties such as; mechanical strength, chemical stability, high electronic conductivity, monodispersed size and high surface area, metal-based NPs have aroused significant interest in electrochemical sensors [26]. To this end, metal NPs such as; gold NPs [94], silver NPs [95], bismuth NPs [96], silica NPs [97], quantum dots [98], amongst others, have found applications in electrode systems as electrochemical sensors for the detection of trace metals in water. The fast electron transfer between the electrochemical transducer and the analyte material in the presence of metal NPs of various size, porosity, conductivity, and shape have been extensively studied. Reports from these studies identified the aggregation of the transition metal-based nanoparticles as a drawback, leading to the clogging of active sites, resulting in the poor reproducibility and sensitivity of the sensors [26,89,93,99]. To address this drawback, support materials including GO [74], graphene [100], graphene carbon nitride (GCN) [89], porous carbon [101], graphene quantum dots [102], and transition metals [103], amongst others, have been incorporated to the metal NPs structure. Discussions on the metal-based nanomaterials for electrochemical sensors are classified into noble and non-noble metal-based nanomaterials in the section below and are also summarized in Table 2.

#### 5.2.1. Noble Metal Based Nanomaterials

Amongst other noble metal-based nanomaterials, gold and silver NPs have been widely employed in electrochemical sensors for the detection of metals in water samples. These nanomaterials exhibit distinct electronic and electrical characteristics, versatile electrocatalytic activity and high surface area. 

##### Gold Nanoparticle-Based Nanocomposites

**Figure 4:**
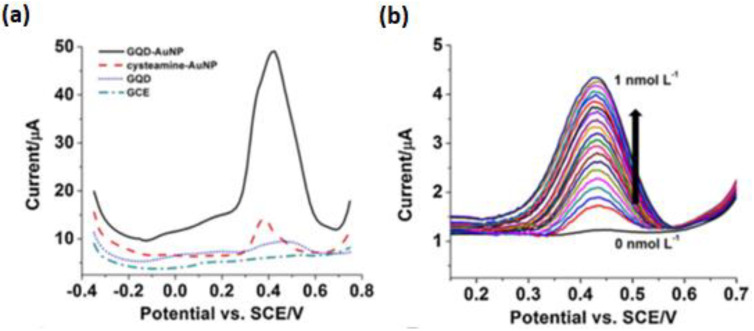
(**a**) Anodic stripping voltammetry (ASV) of graphene quantum dots-gold nanoparticles (GQD-AuNP) modified with GCE in the presence of 0.1 mM of Hg^2+^; (**b**) ASV curves of GQD-AuNP electrode with various concentrations of Hg^2+^ (reproduced with permission from [102], copyright 2015 Elsevier Ltd.).

Gold nanoparticles (AuNPs) have attracted momentous interest in electrochemical devices, particularly in electrochemical sensors, due to their interesting physical and chemical properties. Amongst these properties, the conductivity of AuNPs allows for the intensive electron transfer between the electrode and the analyte of interest [94,102]. Due to their stable uniform surface capable of further functionalization leading to their increased sensitivity and selectivity, AuNPs of small size are used in electrochemical sensors [104]. It has been demonstrated that hybridizing AuNPs with conductive materials such as; rGO [105] and nitrogen-doped graphene [106], amongst others, can enhance the performance for the electrochemical detection. However, such hybridization often involves immobilizing the AuNPs with aptamers [107] or capping the NPs with capping agents in order to reduce the agglomeration of the nanoparticles [108]. To demonstrate this, Ting et al. [102] functionalized cysteamine-capped AuNPs with graphene quantum dots (GQDs) for the electrochemical detection of Hg^2+^ and Cu^2+^ with LODs of 0.02 and 0.05 nM, respectively, using the anodic stripping voltammetry (ASV) method. The sensor showed high sensitivities of 2.47 and 3.69 μA/nM for Hg^2+^ and Cu^2+^, respectively. The high performance of the electrochemical sensor was attributed to the synergistic effect between the highly electron conductive GQDs and cysteamine-capped AuNPs that enhanced the sensitivity of the sensor, as shown in Figure 4. It can be clearly perceived that the integration of GQD on the cysteamine-AuNP dramatically enhanced the signal in Figure 4A. The results for the detection of Hg^2+^ (Figure 4B) were also consistent, following the successful integration of GQD with cysteamine-AuNP. 

##### Silver Nanoparticle-Based Nanocomposites

Silver nanoparticles (AgNPs) are one of the most vital NPs characterized with unique optical, electrical, and thermal properties. Additionally, AgNPs provide excellent electron transfer rate and good chemical stability that is dependent on the size uniformity and distribution across the surface [109]. Through the integration with conductive carbon substrates such as; carbon screen-printed electrodes (CSPE) [110], rGO [111], and other materials such as; AuNPs [112] and SiO [113], AgNPs have shown an increase in sensitivity when applied as electrochemical sensors. However, just like their counterpart AuNPs, aptamers [114] or capping agents [115] are often immobilized prior to doping. To illustrate this, Eksin et al. [95] reported an electrochemical sensor based on AgNPs capped with folic acid modified with a pencil graphite electrode for the detection of Hg^2+^ in tap water samples. The authors reported that folic acid protects the AgNPs from aggregation via binding the amine groups with AgNPs, allowing the folic acid to stabilize the surface of AgNPs by electrostatic repulsion. Under optimized conditions, the linear range and the LOD were obtained as 10–25 µM and 8.43 µM, respectively. Additionally, the sensor was found to be rapid, selective, and sensitive towards monitoring of Hg^2+^ in environmental tap water samples.

#### 5.2.2. Non-Noble Metal-Based Nanomaterials

##### Bismuth Based Nanocomposites

**Figure 5:**
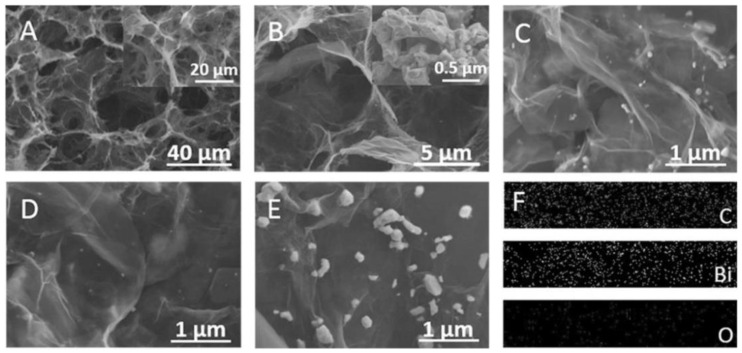
FESEM images of (**A**) 3D graphene framework (GF)/BiNP film, inset: 3D GF film, (**B**) graphene framework of GF/BiNP film, inset: pure BiNPs prepared in a homogenous solution, (**C**) uniformly dispersive BiNPs on graphene surface prepared with 2 mg mL^−1^ Bi(NO_3_)_3_, BiNPs on graphene prepared with (**D**) 0.5 mg mL^−1^ and (**E**) 4 mg mL^−1^ Bi(NO_3_)_3_. (**F**) EDX mappings of C, Bi and O element distribution respectively. (Reproduced with permission from [100], Copyright 2017 Elsevier B.V).

Though bismuth NPs exhibit the same crystal structure as bulk bismuth, their unique physical and chemical properties, such as; a large surface area and surface free energy, play a significant role in electrochemical applications [116]. The drawback on the application of Bi-based NPs as electrochemical nanosensors is their tendency to easily aggregate, as a result of their high surface energy observed leading to low sensor performance [117]. Interestingly, the physicochemical properties of bismuth nanoparticles have been improved using various strategies involving the integration with carbon nanomaterials such as; graphene, CNTs, graphite nanofiber, and metal–organic frameworks (MOFs)-derived porous carbons, leading to an improved sensor performance [96,117]. For this purpose, Shi et al. [100] dispersed bismuth NPs on an electrochemically reduced porous 3D graphene framework to form a 3D graphene framework/Bi nanoparticle (GF/BiNP) film. The film showed a wide linear range from 1 to 120 µg L^−1^ and ultralow LOD of 0.02 µg L^−1^ and 0.05 µg L^−1^ for Pb^2+^ and Cd^2+^, respectively, alongside the linear range from 40 to 300 µg L^−1^ and a low LOD of 4.0 µg L^−1^ of Zn^2+^. In addition to the excellent repeatability, reproducibility and stability, the sensor displayed 98–101% recovery with a % RSD of below 6% when applied in lake and tap water. The performance of the sensor was ascribed to the large active surface area, fast electron transfer ability, high mass transfer efficiency, excellent structure stability and binding strength on the electrode emancipating from the film’s porous structure, as shown in the FESEM images in Figure 5. The incorporation of Bi NPs onto the graphene networks also played a role in the fast electron transfer ability due to an increase in the conductivity. In another study, Chen et al. [101] fabricated BiSn alloy NPs supported on carbon black using the co-precipitation process for the sensitive detection of Cd^2+^ in river water samples. After the differential pulse anodic stripping voltammetry (DPASV) analysis, the fabricated BiSn@C-based sensor showed improved performance on the detection of Cd^2+^, which was ascribed to the large specific surface area, abundance of active sites and good electrical conductivity. These properties were observed to have increased through the addition of carbon black as a support for BiSn, thus increasing the amount of surface-active BiSn, leading to an improved electrochemical performance than BiSn without carbon black. In addition to the high sensitivity, reproducibility, linearity and low LOD, the sensor displayed good recoveries of 94.3–100.8% for Cd^2+^ in river water samples.

### 5.3. Inorganic Non-Metallic Nanomaterials

Inorganic non-metallic nanomaterials are a class of nanomaterials other than polymers and metallic nanomaterials. This class encompasses oxides, carbides, nitrides, halogen compounds, silicates, aluminate, phosphates and borates, amongst other substances [118,119,120]. The identified major types of inorganic non-metallic nanomaterials that include silica- and quantum dot-based nanocomposites are discussed here.

#### 5.3.1. Silica-Based Nanocomposites

Considering their interesting surface chemistry, allowing their surface to be easily functionalized, silica NPs, particularly mesoporous silica NPs, have gained interest in electrochemical reactions [118]. Interesting properties such as; high surface area, diverse structure and morphology have invoked their applications in the agricultural field, food industry, drug delivery, and industrial applications, particularly for the environmental pollution assessments [118,119]. To improve the conductivity and the reduction in the agglomeration of silica nanomaterials, Fang et al. [120] developed the three-dimensional nanoporous silica incorporated with polyfurfural on the GCE by one-step co-electropolymerization for the simultaneous detection of Cd^2+^ and Pb^2+^ in portable water. The sensor displayed a wide linear range of 1.5 ng/L to 6200 μg/L and 0.05 ng/L to 5200 μg/L, with corresponding low LODs of 0.9 ng/L and 0.02 ng/L for Cd^2+^ and Pb^2+^, respectively. Fabricating polyfurfural into nanoporous silica enhanced the sensor performance through improving the electrode surface area, leading to a minimal agglomeration of the nanocomposite and good electrical conductivity through the supply of more binding sites for coordinating with metal ions. Due to this, the 3D-PF/SA showed a higher electrochemical performance than the pristine polyfurfural film modified GCE or pristine nanoporous silica on GCE. 

#### 5.3.2. Quantum Dot-Based Nanocomposites

Although semiconductor nanostructures such as; quantum dots are well-known effective nanomaterials for optical sensors, their use in electrochemical sensors has been shown to be equally effective [97,103]. Quantum dots exhibit distinctive electronic and electrochemical properties leading to high sensitivity, efficient electron transfer, fast response time and long life time [121]. Due to these properties, SnO_2_, ZnO and N-doped carbon quantum dot-based electrochemical sensors, amongst others, were prepared for the detection of Cd [98], Hg [122], Pb [123] in environmental water samples, respectively. Interestingly, Bakhsh et al. [124] fabricated an effective core-shell nanocomposite based on ZnSe–CdSe nanoparticles coated with silica (SiO_2_) nanomaterials using the sol-gel method for the electrochemical detection of Cu^2+^ in water samples. The FESEM images showed the morphology of ZnSe–CdSe core NPs with SiO_2_ shell surrounding the NPs, which were in agreement with the XPS results. The amperometric sensor based on ZnSe–CdSe/SiO_2_ core–shell nanomaterial modified electrode exhibited the LOD of 50 µg L^−1^ and a sensitivity of 0.008847 µA µg^−1^cm^−2^. Additionally, the electrochemical sensor displayed good reproducibility and stability for long term deployment. 

Table 2 represents the selected transition metal/non-metal materials modified with various polymers (chitosan), organic-inorganic hybrid materials (MOFs) and their corresponding analytical performances. The modification of Fe with chitosan leading to the formation of the Fe-chitosan/SPCE nanocomposite has been studied. Compared to the bare electrode, the electrode modified with Fe-chitosan showed an increase in electroanalytical performance with an increased sensitivity, a low LOD on a wide linear range with a low % RSD < 5% towards the detection of As^3+^ (Table 2). The increased performance was due to the selective interaction between Fe-chitosan and As(III) due to the presence of –OH and –NH_2_ groups in the biopolymer which provide adsorption sites for As(III), the enhanced conductivity as the result of the presence of Fe in Fe-chitosan nanocomposite and the enhanced electrochemically active surface areas. Additionally, the sensor also displayed high selectivity, reproducibility, and stability. The modified NPBiE/CE nanocomposite displayed better electroanalytical performance than the conventional NPBiE with higher sensitivities and LODs with a lower % RSD towards the detection of Cd^2+^ and Pb^2+^ (Table 2). The improved performance was due to the modified fabrication process (subsequent steps of Bi and Sn electroplating and heat treatment for Bi-Sn alloying) providing a better structure formation of a well-defined and uniformly distributed Bi nanoporous surface with ellipse, thus improving sensor sensitivity toward heavy metals. The sensor also showed better reproducibility and stability. Then, the ZIF-67/EG nanocomposite was constructed and the electrochemical sensor displayed good performance with a large electrochemically active surface area, high sensitivity and low LOD at low % RSD (Table 2). The improved sensor performances originate from the synergistic effects originating from the intercalation of ZIF-67 in between the layers of EG, allowing for the increased electron conductivity of the nanocomposite. Furthermore, the large BET surface area and pore structure result in increased electrochemical activity, which is beneficial for metal adsorption and diffusion. Additionally, good experimental reproducibility and good stability of the nanocomposite enable the long-term use for selective detection of trace metals (Table 2) in aqueous solutions. Interestingly, the modification of CPSPE with AuNSs in the developed AuNSs/CSPE nanocomposite was observed to improve not only the sensitivity and LODs at a low % RSD (Table 2), but also the stability of the sensor towards As(III) and Hg(II) detection. The different responses for As(III) and Hg(II) (at high loadings) indicate that nanoparticle shape can greatly influence the analytical response. The Cu-based MOFs/GCE nanocomposite displayed increased sensitivity and LODs at low % RSD (Table 2), which was attributed to the large surface area and abundant active sites in MOFs produced a multiplex ratiometric electrochemical sensor with improved characteristics ranging from the reproducibility to the stability of the sensor.

**Table 2:** Electroanalytical performances of selected transition metal-based nanocomposites as nanosensors for trace metal detection in water matrices.

Nanomaterial/Electrode	Synthesis Method	Target Analyte	Electrochem Method	LOD	Linear Range	RSD (%)	n	Sensitivity	Ref
**Fe-Chitosan/CE**	Electrodeposition	As^3+^	SWASV	1.12 ppb	2–24 ppb	2.90	8	3.66 µA/ppb	[125]
**Modified nanoporous bismuth film/CE**	co-electroplating	Cd^2+^	SWASV	1.30 (ppb)	2–40 (ppb)	3.10	40	0.035 (µA/ppb)	[96]
		Pb^2+^		1.50 (ppb)		4.30		0.125 (µA/ppb)	
**ZIF-67/EG**	One-pot hydrothermal reaction	Cd^2+^	SWASV	1.13 (nM)	500–3000 (ppt)	-	-	-	[126]
		Pb^2+^		1.11 (nM)					
		Cu^2+^		2.23 (nM)					
		Hg^2+^		1.28 (nM)					
**Gold nanostars (AuNSs)/CE**	Chemical reduction	As^3+^	SWASV	0.80 (ppb)	2.5–764.2 (ppb)	2.50	3	-	[94]
		Hg^2+^		0.50 (ppb)	1.5–538.9 (ppb)	3.20			
		Pb^2+^		4.30 (ppb)	13.0–323.6 (ppb)	4.60			
**Cu based MOFs/GCE**	Co-precipitation	Cd^2+^	Ratiometric DPV	33.0 (nM)	10 nM to 10 μM	2.21	5	-	[127]
		Pb^2+^		50.0 (nM)	10 nM to 10 mM				

n = reproducibility, CE = carbon electrode, GCE = glassy carbon electrode, EG = expanded graphite.

### 5.4. Nanostructured Metal Oxides/Hydroxides

Nanostructured metal oxides/hydroxides possess significant electron-transfer kinetics, a larger specific surface area, unique electrochemical activity, stability, and a larger number of adsorption sites [24]. Due to their excellent electrochemical properties, nanostructured transition metal oxides/hydroxides have been utilized as sensing elements in numerous electrochemical sensing applications [128,129]. However, nanostructured metal oxides/hydroxides nanomaterials easily aggregate due to their high surface energy, resulting in a significant decrease in accessible catalytic sites [130]. Nonetheless, tailoring the morphology of metal oxides/hydroxides to reduce agglomeration improves the surface area and surface functionality, leading to improved electrochemical performance [1]. Amongst various strategies developed to overcome this problem, one key solution is to support metal oxides on conductive materials, such as; nanostructured carbon materials [69] and metal NPs [45], amongst others. 

#### 5.4.1. Fe_3_O_4_-Based Nanocomposites

**Scheme 3:**
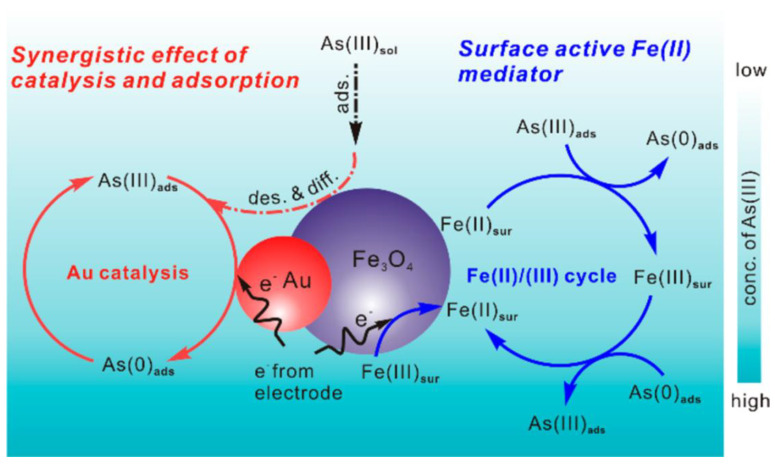
Ultra-highly sensitive electroanalysis of As(III) based on the adsorption of ~10 nm Fe_3_O_4_ nanoparticles (NPs) and the catalyst of ~7 nm Au NPs as well as the redox mediation by surface-active Fe(II) at Au/Fe_3_O_4_ SPCE (Reproduced with permission from [135], Copyright 2018 American Chemical Society).

Fe_3_O_4_ nanomaterials possess a high adsorption capacity, which renders them suitable for the electrochemical detection of metals [131]. Although the oxidation of surface Fe^2+^ ions in Fe_3_O_4_ nanomaterials allows for the effortless reduction of metal cations, the conductivity of Fe_3_O_4_ nanomaterials still needs to be improved, alongside the de-aggregation of the metal NPs [132,133]. In response to this, Wu et al. [134] supported Fe_3_O_4_ NPs on the highly conductive fluorinated-MWCNT using a hydrothermal method for the simultaneous detection of Cd^2+^, Pb^2+^, Cu^2+^, and Hg^2+^ in water samples. Astonishingly, the Fe_3_O_4_/F-MWCNT-based electrochemical sensor displayed the best performance, with a sensitivity of 108.79, 125.91, 160.85, and 312.65 μA mM^−1^ cm^−2^, wider linear detection ranges of 0.5–30.0, 0.5–30.0, 0.5–30.0, and 0.5–20.0 μM, and lower LODs of 0.05, 0.08, 0.02, and 0.05 nM, towards Cd^2+^, Pb^2+^, Cu^2+^, and Hg^2+^, respectively. In addition to displaying higher recoveries of 97–101% in river water samples, the electrochemical sensor exhibited excellent performances in selectivity, recovery, stability, and reproducibility studies. The excellent performance could be attributed to the semi-ionic C-F bond on the F-MWCNTs’ surface with a strong negative charge and the synergistic interaction between Fe_3_O_4_ and F-MWCNTs. In another study, Li et al. [135] reported on the electrochemical sensing of As(III) using hydrothermally prepared dumbbell-like Au/Fe_3_O_4_ NPs modified with a screen-printed carbon electrode. Interestingly, the synergistic effect of the excellent catalytic properties of Au NPs and the good adsorption ability coupled with an electron hopping process between the Fe^2+^ and Fe^3+^ ions in Fe_3_O_4_ NPs significantly improve the detection of As(III). The mechanism of detection (Scheme 3) involved the adsorption of As(III) on Fe_3_O_4_ NPs, whereby the surface active Fe(II) is oxidized to Fe(III) simultaneously with the reduction of As(III) to As(0). However, during the SWASV process on the electrode surface, Fe(III) is reduced back to Fe(II) with the oxidation of As(0) to As(III) to complete the Fe(II)/Fe(III) cycle. The adsorbed As(III) on Fe_3_O_4_ NPs is then directly reduced and oxidized on the Au surface with an increased sensitivity of 9.43 μA ppb^−1^ and a low LODs of 0.0215 ppb in the 0.1−10 ppb linear range. Good recoveries of 90−110% were observed for the analysis of river water.

#### 5.4.2. Layered Double Hydroxide-Based Nanocomposites

Layered double hydroxides (LDHs) are a family of two-dimensional (2D) materials with layered crystal structures consisting of metal cations occupying the center of the edge sharing octahedral unit, with vertexes containing hydroxide ions that make it possible to form 2D sheets [136]. The chemical composition of LDH can be shown with a generic formula of the most studied class of LDH, expressed as [M^2+^_1−x_ M^3+^_x_ (OH)_2_]^x+^ [A^n−^_x/n_]^x-^ . zH_2_O, where M^2+^ = divalent cation, M^3+^ = trivalent cation, A = interlayer anion, n = charge on interlayer anion, x and z are fractional constants. The common divalent cations used in LDH include Ca^2+^, Mg^2+^, Mn^2+^, Fe^2+^, Co^2+^, Ni^2+^, Cu^2+^ or Zn^2+^, whereas the common trivalent cations used in LDH include Fe^3+^, Al^3+^, Ga^3+^, Mn^3+^ and Co^3+^. A variety of anions are used to compensate for the positive charges of the brucite layers, including CO_3_^2−^, Cl^−^, SO_4_^2−^, and RCO^2−^ [136,137,138,139]. The synthesis of LDH has been carried out using top down and bottom up methods [138,139]. Top down synthesis methods include the delamination/exfoliation step in solvents such as; butanol, acrylates, toluene and tetrachloromethane, formamide, dimethylformamide (DMF)–ethanol mixture and water [138,140,141]. Bottom up methods follow the traditional co-precipitation method [139]. Due to their many advantages, such as; high adsorption capacity, exchange-able interlayer anions, ease of preparation, catalytic activity and low cost, LDHs have been previously synthesized with a wide variety of morphologies, from discrete 2D nanosheets to nanoflowers [136,138,139]. These LDHs have found applications in catalysis, fire retardation, and sensors, amongst other applications. Asadpour-Zeynali and Amini [142] developed an electrochemical sensor based on thioglycolic acid (TGA) intercalated Mg–Al LDH (Mg–Al–TGA LDH) using the anion exchange method for the selective detection of trace mercury ions using SWASV. Under optimal conditions, the sensor exhibited a wider linearity range from 2.0 to 800 nM Hg(II) with the LOD of 0.8 nM. The TGA intercalated between the Mg-Al layers, confirmed by the increase in the interlayer basal space regions on the XRD, provided strong chelating thiol groups towards Hg (II), thus exhibiting excellent performance with a percentage recovery ranging from 97 to 104%. 

Table 3 represents the modifications of transition metal oxides/hydroxides alongside their resulting analytical performance. Transition metal oxides/hydroxides have been modified with various classes of materials ranging from nanostructured carbon, metal NPs, polymers, amino acids, organic compounds, metal oxides and organic-inorganic hybrids. To this end, the highly modified Fe_3_O_4_/MWCNTs/LSG/CS/GCE nanocomposite displayed increased sensitivity and LOD at a low % RSD after modifications, as shown in Table 3. As expected, the novel nanocomposite possessed a significantly large specific area, remarkably high conductivity and excellent absorption ability that assumed responsibility for the increase in the analytical performance of the sensor. Additionally, the sensor displayed excellent repeatability, reproducibility, stability, and reliable practical applicability. The CeO_2_-CNF nanocomposite was applied for the detection of Pb and Cu, and the electrochemical analysis indicated the high sensitivity and lower LOD with low % RSD (Table 3). The increased analytical performance was attributed to the high BET surface area and the graphitic character due to the presence of the CNF, which was responsible for the rapid electron transport from the solution to the electrode surface. The reproducibility and linearity of the sensor were found to also be higher. The modification of MnO_2_ by MWCNTs/CS resulted in the MnO_2_/MWCNT/CS nanocomposite that showed higher sensitivity and low LOD at low % RSDs (Table 3) when applied on the detection of Cr(III). The improved electrochemical performance was due to the porous microstructure and the reduced agglomeration of manganese oxide nanostructures, and the high ability of chitosan for encapsulation of more crystalline MnO_x_, with MWCNTs/CS as a good dispersant agent leading to homogenous particles that were highly stable. The synergistic effects of MWCNTs and MnO_x_ nanoflakes played an important role on the stability and increased performance of the sensor. The Fe-MOF@mFe_3_O_4_@mC nanocomposite demonstrated excellent electrochemical activity with high sensitivity, good linear range, and low LOD at a low % RSD (Table 3) for the detection of Pb^2+^ and As^3+^. The strong biobinding of the aptamer with the metal ion played a key role in increasing the sensitivity, selectivity, stability, and reproducibility of the sensor. The ZnO nanofibers/L-cysteine nanocomposite/GCE displayed a wide linear range, increased sensitivity, low LOD at a low % RSD (Table 3) on the detection of Pb^2+^. The high performance of the sensor was ascribed to the high porosity of nanofibers combined with the Lewis acid–base interaction of L-cysteine. The sensor displayed satisfying stability, excellent reproducibility, and good repeatability. The BiNPs@CoFe_2_O_4_/GCE nanocomposite showed impressive analytical responses with high sensitivity, wide linear range, and low LOD at low % RSD on the detection of Pb^2+^ and Cd^2+^ as shown in Table 3. The high performance of the sensor was ascertained as being due to the excellent adsorption and electrical conductivity that contributed to the acceptable reproducibility and good stability exhibited by the sensor. The Co_3_O_4_ nanosheets/ITO nanocomposite formed by fabricating Co_3_O_4_ nanosheets on ITO showed good electrochemical performance with a wide linear range and low LOD at low % RSD (Table 3). The improved performance was dedicated to the presence of Co_3_O_4_ nanosheets on the electrode surface that could enhance the electrochemical activity of the electrode. The sensor also displayed good selectivity, sensitivity and reproducibility.

## 6. Smart Electrochemical Nanosensors

The fourth industrial revolution has evoked the development of cognitive system/devices and their exploitation in various technical fields [149,150]. Although smart devices such as; smartphones, medical, robotics and automobile devices have revolutionized industrial technologies, the emerging smart electrochemical environmental nanosensing technology is expected to have an equally positive impact on the industrial revolution [149,151]. The smart electrochemical environmental nanosensing technology is not only associated with the nanosensor device (recognition element, transducer and the readout system), but is integrated with add on components such as; light-emitting diodes (LEDs), display screens, microprocessors, wireless communication boards (Bluetooth, infrared or Wi-Fi) and power supply battery/solar devices, thus enabling the processing/display to be performed remotely far from the sensing site [149,150,151,152]. The most essential part of the smart nanosensor is the recognition element, allowing for the recognition of the analyte of interest [153]. Engineering the materials involved in the recognition systems is therefore the foundation leading to the smartness of the sensing devices. To this end, strategies leading to the maximization of the performance of nanomaterials involved as recognizing elements for trace metal detection in the environment, particularly in water matrices, are essential to the development of the emerging smart electrochemical nanosensor technology. Research based on smart electrochemical nanosensors for trace metal detection in water is still at a developmental stage and therefore more work still needs to be done at a laboratory scale prior to upscaling into the industrial scale. 

## 7. Summary and Future Prospects

The recent advancements on the design and development of nanocomposites as nanosensors for trace metals detection in environmental water matrices were systematically reviewed. Following a brief introduction on the nanosensors, important parameters for evaluating their performance including sensitivity, selectivity, limit of detection, linearity, response time, reproducibility, repeatability and stability were outlined. Then, three major strategies involved in the design of the nanocomposites, including engineering the active sites, engineering the conductivity and creating a porous structure, were realized. The studies reviewed here show that these strategies were achieved by integrating nanomaterials of three different classes that included carbon nanostructures, transition metal and transition metal hydroxides/oxides nanomaterials and applying them for trace metal detection in water matrices as summarized in Table 1, Table 2 and Table 3. These integrations could reduce the aggregations, π−π stacking interactions, agglomerations, low conductivity, and low stability attained, amongst other challenges. The applications of the nanocomposites on the detection of various trace metals in various water matrices were also reviewed and discussed. Notwithstanding all the work done on nanosensors for environmental applications, there are still some challenges that still need to be solved prior to the widespread application and deeper understanding of the mechanisms involved. Furthermore, the optimizations of the post-treatment methods such as; thermal treatment and exfoliation techniques could improve the performance of the nanosensors and enhance their 3S (stability, sensitivity and stability). The current developments on smart electrochemical nanosensors integrating nanosensors with smart systems for environmental applications have also been reviewed. Although these systems are still at an infant stage, their impact on revolutionizing sensor systems is expected to be positive. Smart electrochemical nanosensors could revolutionize the sensor technology as they are aimed at solving previous detected challenges facing nanosensors. These include(s) their deployment in previously inaccessible areas, integrating many nanosensors devices and monitor their response remotely far from sites of interest and frequently monitoring large areas of interest, including both upstream and downstream, enabling the tracking of the sources of any developing trace metal discharges.

## Figures and Tables

**Table 3:** Electroanalytical performances of selected transition metal oxide/hydroxide-based nanocomposites as sensors for trace metal detection in water matrices.

Nanomaterial/Electrode	Synthesis Method	Target Analyte	Electrochem Method	LOD	Linear Range	RSD (%)	n	Sensitivity	Ref
**Fe_3_O_4_/MWCNTs/LSG/CS/GCE**	Hydrothermal/laser scribing	Cd^2+^	SWASV	0.10 ppb	1 to 200 ppb	3.77	10	-	[143]
		Pb^2+^		0.07 ppb		0.97			
**CeO_2_-CNF**	chemical vapor deposition	Pb^2+^	DPV	0.60 ppb	0.9–2.1 ppb	-	-	-	[144]
		Cu^2+^		0.30 ppb	0.6–1.8 ppb				
**MnO_2_/MWCNT/CS**	Sonochemical/Electrodeposition	Cr^3+^	HC	0.30 ppb	3 to 200 ppb	3.00	5	18.7 nA µM^−1^ cm^−2^	[145]
**Fe-MOF@mFe_3_O_4_@mC**	Hydrothermal/temperature treatment	Pb^2+^	EIS	2.27 pM	0.01 to 10.0 nM	5.61	5	-	[146]
		As^3+^		6.73 pM		4.66			
**ZnO nanofibers/L-cysteine nanocomposite/GCE**	Electrospinning/annealing	Pb^2+^	SWASV	0.40 ppb	10–140 ppb	4.50	30	-	[147]
**BiNPs@CoFe_2_O_4_/GCE**	Hydrothermal reaction/colloidal dispersion	Cd^2+^	SWASV	7.30 nM	0.06 to 0.6 mM	3.20	10	-	[99]
		Pb^2+^		8.20 nM	0.08 to 0.8 mM	2.50			
**Co_3_O_4_ nanosheets/ITO**	Electrodeposition	Pb^2+^	DPASV	0.52 ppb	1–100 ppb	5.20	7	-	[148]

LOD = limit of detection, RSD = relative standard deviation, n = reproducibility, CE = carbon electrode, CS = chitosan, GCE = glassy carbon electrode, ITO = indium tin oxide, LSG = laser scribed graphene, mC = mesoporous carbon, HC = hydrodynamic chronoamperometry.

## Data Availability

Data sharing not applicable.

## References

[1] Akanji S.P., Ama O.M., Ray S.S., Osifo P.O. (2020). Metal Oxide Nanomaterials for Electrochemical Detection of Heavy Metals in Water. Nanostructured Metal-Oxide Electrode Materials for Water Purification.

[2] Sihlahla M., Mouri H., Nomngongo P.N. (2019). Uptake of trace elements by vegetable plants grown on agricultural soils: Evaluation of trace metal accumulation and potential health risk. J. Afr. Earth Sci..

[3] Donner M.W., Arshad M., Ullah A., Siddique T. (2019). Unravelled keratin-derived biopolymers as novel biosorbents for the simultaneous removal of multiple trace metals from industrial wastewater. Sci. Total Environ..

[4] Embaby A., Redwan M. (2019). Sources and behavior of trace elements in groundwater in the South Eastern Desert, Egypt. Environ. Monit. Assess..

[5] Strzelec M., Proemse B.C., Barmuta L.A., Gault-Ringold M., Desservettaz M., Boyd P.W., Perron M.M.G., Schofield R., Bowie A.R. (2020). Atmospheric Trace Metal Deposition from Natural and Anthropogenic Sources in Western Australia. Atmosphere.

[6] Munonde T.S., Maxakato N.W., Nomngongo P.N. (2017). Preconcentration and speciation of chromium species using ICP-OES after ultrasound-assisted magnetic solid phase extraction with an amino-modified magnetic nanocomposite prepared from Fe_3_O_4_, MnO_2_ and Al_2_O_3_. Microchim. Acta.

[7] Jakavula S., Biata N.R., Dimpe K.M., Pakade V.E., Nomngongo P.N. (2020). A Critical Review on the Synthesis and Application of Ion-Imprinted Polymers for Selective Preconcentration, Speciation, Removal and Determination of Trace and Essential Metals from Different Matrices. Crit. Rev. Anal. Chem..

[8] Bocca B., Ruggieri F., Pino A., Rovira J., Calamandrei G., Martínez M.Á., Domingo J.L., Alimonti A., Schuhmacher M. (2019). Human biomonitoring to evaluate exposure to toxic and essential trace elements during pregnancy. Part A. concentrations in maternal blood, urine and cord blood. Environ. Res..

[9] Nnorom I.C., Ewuzie U., Eze S.O. (2019). Multivariate statistical approach and water quality assessment of natural springs and other drinking water sources in Southeastern Nigeria. Heliyon.

[10] Cotruvo J.A. (2017). 2017 WHO Guidelines for Drinking Water Quality: First Addendum to the Fourth Edition. J. Am. Water Work. Assoc..

[11] Khound N.J., Phukon P., Bhattacharyya K.G. (2019). Toxic Trace Metals in the Surface Water Sources of Jia–Bharali river basin, North Brahmaputra Plain, India—A Hydrochemical Elucidation. Water Resour..

[12] Munonde T.S., Maxakato N.W., Nomngongo P.N. (2018). Preparation of magnetic Fe_3_O_4_ nanocomposites modified with MnO_2_, Al_2_O_3_, Au and their application for preconcentration of arsenic in river water samples. J. Environ. Chem. Eng..

[13] Filik H., Avan A.A. (2017). Ionic Liquid Based Dispersive Liquid-Liquid Microextraction Combined with Magnetic-Based Dispersive Micro-Solid-Phase Extraction for Determination of Trace Cobalt in Water Samples by FAAS. Curr. Anal. Chem..

[14] Han Q., Huo Y., Yang L., Yang X., He Y., Wu J. (2018). Determination of Trace Nickel in Water Samples by Graphite Furnace Atomic Absorption Spectrometry after Mixed Micelle-Mediated Cloud Point Extraction. Molecules.

[15] Zhou S., Yuan Z., Cheng Q., Zhang Z., Yang J. (2018). Rapid in situ determination of heavy metal concentrations in polluted water via portable XRF: Using Cu and Pb as example. Environ. Pollut..

[16] Wang J., Wu S., Suo X.-K., Liao H. (2019). The Processes for Fabricating Nanopowders. Advanced Nanomaterials and Coatings by Thermal Spray.

[17] Kokab T., Shah A., Nisar J., Khan A.M., Khan S.B., Shah A.H. (2020). Tripeptide Derivative-Modified Glassy Carbon Electrode: A Novel Electrochemical Sensor for Sensitive and Selective Detection of Cd^2+^ Ions. ACS Omega.

[18] Munir A., Shah A., Nisar J., Ashiq M.N., Akhter M.S., Shah A.H. (2019). Selective and simultaneous detection of Zn^2+^, Cd^2+^, Pb^2+^, Cu^2+^, Hg^2+^ and Sr^2+^ using surfactant modified electrochemical sensors. Electrochim. Acta.

[19] Wang Y., Zhao S., Li M., Li W., Zhao Y., Qi J., Cui X. (2017). Graphene quantum dots decorated graphene as an enhanced sensing platform for sensitive and selective detection of copper(II). J. Electroanal. Chem..

[20] Baig N., Sajid M., Saleh T.A. (2019). Recent trends in nanomaterial-modified electrodes for electroanalytical applications. TrAC Trends Anal. Chem..

[21] Oliveira T.M.B.F., Morais S. (2018). New Generation of Electrochemical Sensors Based on Multi-Walled Carbon Nanotubes. Appl. Sci..

[22] Munawar A., Ong Y., Schirhagl R., Tahir M.A., Khan W.S., Bajwa S.Z. (2019). Nanosensors for diagnosis with optical, electric and mechanical transducers. RSC Adv..

[23] Li Y., Chen Y., Yu H., Tian L., Wang Z. (2018). Portable and smart devices for monitoring heavy metal ions integrated with nanomaterials. TrAC Trends Anal. Chem..

[24] Khan I., Saeed K., Khan I. (2019). Nanoparticles: Properties, applications and toxicities. Arab. J. Chem..

[25] Wu Z., Yang S., Wu W. (2016). Shape control of inorganic nanoparticles from solution. Nanoscale.

[26] Maduraiveeran G., Jin W. (2017). Nanomaterials based electrochemical sensor and biosensor platforms for environmental applications. Trends Environ. Anal. Chem..

[27] Huang H., Chen L., Wang S., Kang P., Chen X., Guo Z., Huang X.-J. (2019). Electrochemical monitoring of persistent toxic substances using metal oxide and its composite nanomaterials: Design, preparation, and application. TrAC Trends Anal. Chem..

[28] Lu Z., Zhang J., Dai W., Lin X., Ye J., Ye J. (2017). A screen-printed carbon electrode modified with a bismuth film and gold nanoparticles for simultaneous stripping voltammetric determination of Zn(II), Pb(II) and Cu(II). Microchim. Acta.

[29] Pérez-Ràfols C., Serrano N., Díaz-Cruz J.M., Ariño C., Esteban M. (2016). New approaches to antimony film screen-printed electrodes using carbon-based nanomaterials substrates. Anal. Chim. Acta.

[30] Zuo Y., Xu J., Zhu X., Duan X., Lu L., Yu Y. (2019). Graphene-derived nanomaterials as recognition elements for electrochemical determination of heavy metal ions: A review. Microchim. Acta.

[31] De Barros A., Constantino C.J.L., Da Cruz N.C., Bortoleto J.R.R., Ferreira M. (2017). High performance of electrochemical sensors based on LbL films of gold nanoparticles, polyaniline and sodium montmorillonite clay mineral for simultaneous detection of metal ions. Electrochim. Acta.

[32] Eranjaneya H., Adarakatti P.S., Siddaramanna A., Malingappa P., Chandrappa G.T. (2018). Citric acid assisted synthesis of manganese tungstate nanoparticles for simultaneous electrochemical sensing of heavy metal ions. Mater. Sci. Semicond. Process..

[33] Jin W., Fu Y., Hu M., Wang S., Liu Z. (2020). Highly efficient SnS-decorated Bi_2_O_3_ nanosheets for simultaneous electrochemical detection and removal of Cd(II) and Pb(II). J. Electroanal. Chem..

[34] Mohamed M.A., El-Badawy F.M., El-Desoky H.S., Ghoneim M.M. (2017). Magnetic cobalt ferrite nanoparticles CoFe_2_O_4_ platform as an efficient sensor for trace determination of Cu(ii) in water samples and different food products. New J. Chem..

[35] Gan X., Zhao H. (2019). Understanding signal amplification strategies of nanostructured electrochemical sensors for environmental pollutants. Curr. Opin. Electrochem..

[36] Chen X., He X., Gao J., Jiang J., Jiang X., Wu C. (2019). Three-dimensional porous Ni, N-codoped C networks for highly sensitive and selective non-enzymatic glucose sensing. Sens. Actuators B Chem..

[37] Chen H., Liang X., Liu Y., Ai X., Asefa T., Zou X. (2020). Active Site Engineering in Porous Electrocatalysts. Adv. Mater..

[38] Liu W., Yin R., Xu X., Zhang L., Shi W., Cao X. (2019). Structural Engineering of Low-Dimensional Metal–Organic Frameworks: Synthesis, Properties, and Applications. Adv. Sci..

[39] Tuantranont A. (2013). Applications of nanomaterials in sensors and diagnostics. Springer Series on Chemical Sensors and Biosensors.

[40] Kranz C. (2019). Carbon-Based Nanosensor Technology.

[41] Wang Z.L. (2012). Self-Powered Nanosensors and Nanosystems. Adv. Mater..

[42] Vikesland P.J. (2018). Nanosensors for water quality monitoring. Nat. Nanotechnol..

[43] Fuertes G., Soto I., Carrasco R., Vargas M., Sabattin J., Lagos C. (2016). Intelligent Packaging Systems: Sensors and Nanosensors to Monitor Food Quality and Safety. J. Sens..

[44] Justino C.I.L., Freitas A.C., Pereira R., Duarte A.C., Santos T.A.P.R. (2015). Recent developments in recognition elements for chemical sensors and biosensors. TrAC Trends Anal. Chem..

[45] Chen Y., Fan Z., Zhang Z., Niu W., Li C., Yang N., Chen B., Zhang H. (2018). Two-Dimensional Metal Nanomaterials: Synthesis, Properties, and Applications. Chem. Rev..

[46] Mourdikoudis S., Pallares R.M., Thanh N.T.K. (2018). Characterization techniques for nanoparticles: Comparison and complementarity upon studying nanoparticle properties. Nanoscale.

[47] Graboski A.M., Martinazzo J., Ballen S.C., Steffens J., Steffens C. (2020). Nanosensors for water quality control. Nanotechnology in the Beverage Industry.

[48] Mahbub T., Hoque M.E. (2020). Introduction to nanomaterials and nanomanufacturing for nanosensors. Nanofabrication for Smart Nanosensor Applications.

[49] Gregorczyk K., Knez M. (2016). Hybrid nanomaterials through molecular and atomic layer deposition: Top down, bottom up, and in-between approaches to new materials. Prog. Mater. Sci..

[50] Ganachari S.V., Banapurmath N.R., Salimath B., Yaradoddi J.S., Shettar A.S., Hunashyal A.M., Venkataraman A., Patil P., Shoba H., Hiremath G.B. (2017). Synthesis techniques for preparation of nanomaterials. Handbook of Ecomaterials.

[51] Lim T.-C. (2016). Nanosensors: Theory and Applications in Industry, Healthcare and Defense.

[52] Jiang H. (2011). Chemical Preparation of Graphene-Based Nanomaterials and Their Applications in Chemical and Biological Sensors. Small.

[53] Justino C.I.L., Rocha-Santos T.A.P., Cardoso S., Duarte A.C. (2013). Strategies for enhancing the analytical performance of nanomaterial-based sensors. TrAC Trends Anal. Chem..

[54] Fan Y.Z., Tang Q., Liu S.G., Yang Y.Z., Ju Y.J., Xiao N., Luo H.Q., Li N.B. (2019). A smartphone-integrated dual-mode nanosensor based on novel green-fluorescent carbon quantum dots for rapid and highly selective detection of 2,4,6-trinitrophenol and pH. Appl. Surf. Sci..

[55] Cheng C., Zhang R., Wang J., Zhang Y., Wen C., Tan Y., Yang M. (2020). An ultrasensitive and selective fluorescent nanosensor based on porphyrinic metal–organic framework nanoparticles for Cu^2+^ detection. Analyst.

[56] Liu J., Pan L., Shang C., Lu B., Wu R., Feng Y., Chen W., Zhang R., Bu J., Xiong Z. (2020). A highly sensitive and selective nanosensor for near-infrared potassium imaging. Sci. Adv..

[57] Shoaie N., Daneshpour M., Azimzadeh M., Mahshid S., Khoshfetrat S.M., Jahanpeyma F., Gholaminejad A., Omidfar K., Foruzandeh M. (2019). Electrochemical sensors and biosensors based on the use of polyaniline and its nanocomposites: A review on recent advances. Microchim. Acta.

[58] Zhou G., Wang Y., Cui L. (2015). Biomedical Sensor, Device and Measurement Systems. Adv. Bioeng..

[59] Leal-Junior A.G., Frizera A., Pontes M.J. (2018). Sensitive zone parameters and curvature radius evaluation for polymer optical fiber curvature sensors. Opt. Laser Technol..

[60] O’Riordan A., Barry S. (2016). Electrochemical nanosensors: Advances and applications. Rep. Electrochem..

[61] Tang L., Li J. (2017). Plasmon-Based Colorimetric Nanosensors for Ultrasensitive Molecular Diagnostics. ACS Sens..

[62] Moosavi S.M., Ghassabian S. (2018). Linearity of Calibration Curves for Analytical Methods: A Review of Criteria for Assessment of Method Reliability. Calibration and Validation of Analytical Methods—A Sampling of Current Approaches.

[63] Tiwari J.N., Vij V., Kemp K.C., Kim K.S. (2016). Engineered Carbon-Nanomaterial-Based Electrochemical Sensors for Biomolecules. ACS Nano.

[64] Bernal E., Guo X. (2014). Limit of detection and limit of quantification determination in gas chromatography. Adv. Gas Chromatogr..

[65] Hare D.J., New E.J., De Jonge M.D., McColl G. (2015). Imaging metals in biology: Balancing sensitivity, selectivity and spatial resolution. Chem. Soc. Rev..

[66] Schroeder V., Savagatrup S., He M., Lin S., Swager T.M. (2018). Carbon Nanotube Chemical Sensors. Chem. Rev..

[67] De A., Chen S., Carlen E.T. (2014). Probe-free semiconducting silicon nanowire platforms for biosensing. Semiconducting Silicon Nanowires for Biomedical Applications.

[68] Rowland C.E., Brown III C.W., Delehanty J.B., Medintz I.L. (2016). Nanomaterial-based sensors for the detection of biological threat agents. Mater. Today.

[69] Mnyipika S.H., Nomngongo P.N. (2017). Square Wave Anodic Stripping Voltammetry for Simultaneous Determination of Trace Hg(II) and Tl(I) in Surface Water Samples Using SnO_2_@MWCNTs Modified Glassy Carbon Electrode. Int. J. Electrochem. Sci.

[70] Ghosh A., Zhang C., Shi S.Q., Zhang H. (2019). High-Temperature Gas Sensors for Harsh Environment Applications: A Review. CLEAN Soil Air Water.

[71] Arduini F., Cinti S., Scognamiglio V., Moscone D. (2020). Nanomaterial-based sensors. Handbook of Nanomaterials in Analytical Chemistry.

[72] Yan Q.-L., Gozin M., Zhao F.-Q., Cohen A., Pang S.-P. (2016). Highly energetic compositions based on functionalized carbon nanomaterials. Nanoscale.

[73] Syama S., Mohanan P.V. (2019). Comprehensive application of graphene: Emphasis on biomedical concerns. Nano-Micro Lett..

[74] Rahman M.T., Kabir M.F., Gurung A., Reza K.M., Pathak R., Ghimire N., Baride A., Wang Z., Kumar M., Qiao Q. (2019). Graphene Oxide–Silver Nanowire Nanocomposites for Enhanced Sensing of Hg^2+^. ACS Appl. Nano Mater..

[75] Rowley-Neale S.J., Randviir E.P., Dena A.S.A., Banks C.E. (2018). An overview of recent applications of reduced graphene oxide as a basis of electroanalytical sensing platforms. Appl. Mater. Today.

[76] Dai H., Wang N., Wang D., Ma H., Lin M. (2016). An electrochemical sensor based on phytic acid functionalized polypyrrole/graphene oxide nanocomposites for simultaneous determination of Cd(II) and Pb(II). Chem. Eng. J..

[77] Tan S.M., Ambrosi A., Chua C.K., Pumera M. (2014). Electron transfer properties of chemically reduced graphene materials with different oxygen contents. J. Mater. Chem. A.

[78] Malhotra B.D., Ali M.A. (2018). Nanomaterials in Biosensors: Fundamentals and Applications. Nanomater. Biosens..

[79] Ambrosi A., Chua C.K., Latiff N.M., Loo A.H., Wong C.H.A., Eng A.Y.S., Bonanni A., Pumera M. (2016). Graphene and its electrochemistry—An update. Chem. Soc. Rev..

[80] Li J., Xu Z., Liu M., Deng P., Tang S., Jiang J., Feng H., Qian D., He L. (2017). Ag/N-doped reduced graphene oxide incorporated with molecularly imprinted polymer: An advanced electrochemical sensing platform for salbutamol determination. Biosens. Bioelectron..

[81] Hassanpoor S., Rouhi N. (2019). Electrochemical sensor for determination of trace amounts of cadmium (II) in environmental water samples based on MnO_2_/RGO nanocomposite. Int. J. Environ. Anal. Chem..

[82] Dong G., Zhang Y., Pan Q., Qiu J. (2014). A fantastic graphitic carbon nitride (g-C_3_N_4_) material: Electronic structure, photocatalytic and photoelectronic properties. J. Photochem. Photobiol. C Photochem. Rev..

[83] Ong W.-J. (2017). 2D/2D Graphitic Carbon Nitride (g-C_3_N_4_) Heterojunction Nanocomposites for Photocatalysis: Why Does Face-to-Face Interface Matter?. Front. Mater..

[84] Liu L., Lv H., Wang C., Ao Z., Wang G. (2016). Fabrication of the protonated graphitic carbon nitride nanosheets as enhanced electrochemical sensing platforms for hydrogen peroxide and paracetamol detection. Electrochim. Acta.

[85] Ding S., Ali A., Jamal R., Xiang L., Zhong Z., Abdiryim T. (2018). An Electrochemical Sensor of Poly (EDOT-pyridine-EDOT)/Graphitic Carbon Nitride Composite for Simultaneous Detection of Cd^2+^ and Pb^2+^. Materials.

[86] Mishra A., Basu S., Shetti N.P., Reddy K.R., Aminabhavi T.M. (2019). Photocatalysis of graphene and carbon nitride-based functional carbon quantum dots. Nanoscale Materials in Water Purification.

[87] Zhang J., Zhu Z., Di J., Long Y., Li W., Tu Y. (2015). A Sensitive Sensor for trace Hg^2+^ Determination Based on Ultrathin g-C_3_N_4_ Modified Glassy Carbon Electrode. Electrochimica Acta.

[88] Zheng Z.X., Wang M., Shi X.Z., Wang C.M. (2019). Palladium Nanoparticles/Graphitic Carbon Nitride Nanosheets-Carbon Nanotubes as a Catalytic Amplification Platform for the Selective Determination of 17α-ethinylestradiol in Feedstuffs. Sci. Rep..

[89] Karthika A., Nikhil S., Suganthi A., Rajarajan M. (2020). A facile sonochemical approach based on graphene carbon nitride doped silver molybdate immobilized nafion for selective and sensitive electrochemical detection of chromium (VI) in real sample. Adv. Powder Technol..

[90] Mahmoudian M.R., Basirun W.J., Alias Y., MengWoi P. (2020). Investigating the effectiveness of g-C_3_N_4_ on Pt/g-C_3_N_4_/polythiophene nanocomposites performance as an electrochemical sensor for Hg^2+^ detection. J. Environ. Chem. Eng..

[91] Akhtar M., Tahir A., Zulfiqar S., Hanif F., Warsi M.F., Agboola P.O., Shakir I. (2020). Ternary hybrid of polyaniline-alanine-reduced graphene oxide for electrochemical sensing of heavy metal ions. Synth. Met..

[92] Yu L., Zhang Q., Yang B., Xu Q., Xu Q., Hu X. (2018). Electrochemical sensor construction based on Nafion/calcium lignosulphonate functionalized porous graphene nanocomposite and its application for simultaneous detection of trace Pb^2+^ and Cd^2+^. Sens. Actuators B Chem..

[93] Lu Z., Dai W., Lin X., Liu B., Zhang J., Ye J., Ye J. (2018). Facile one-step fabrication of a novel 3D honeycomb-like bismuth nanoparticles decorated N-doped carbon nanosheet frameworks: Ultrasensitive electrochemical sensing of heavy metal ions. Electrochim. Acta.

[94] Dutta S., Strack G., Kurup P. (2019). Gold nanostar electrodes for heavy metal detection. Sens. Actuators B Chem..

[95] Eksin E., Erdem A., Fafal T., Kıvçak B. (2019). Eco-friendly Sensors Developed by Herbal Based Silver Nanoparticles for Electrochemical Detection of Mercury (II) Ion. Electroanalysis.

[96] Hwang J.-H., Wang X., Zhao D., Rex M.M., Cho H.J., Lee W.H. (2019). A novel nanoporous bismuth electrode sensor for in situ heavy metal detection. Electrochim. Acta.

[97] Afkhami A., Soltani-Felehgari F., Madrakian T., Ghaedi H., Rezaeivala M. (2013). Fabrication and application of a new modified electrochemical sensor using nano-silica and a newly synthesized Schiff base for simultaneous determination of Cd^2+^, Cu^2+^ and Hg^2+^ ions in water and some foodstuff samples. Anal. Chim. Acta.

[98] Bhanjana G., Dilbaghi N., Kumar R., Umar A., Kumar S. (2015). SnO_2_ quantum dots as novel platform for electrochemical sensing of cadmium. Electrochim. Acta.

[99] He Y., Wang Z., Ma L., Zhou L., Jiang Y., Gao J. (2020). Synthesis of bismuth nanoparticle-loaded cobalt ferrite for electrochemical detection of heavy metal ions. RSC Adv..

[100] Shi L., Li Y., Rong X., Wang Y., Ding S. (2017). Facile fabrication of a novel 3D graphene framework/Bi nanoparticle film for ultrasensitive electrochemical assays of heavy metal ions. Anal. Chim. Acta.

[101] Chen Y., Zhang D., Wang D., Lu L., Wang X., Guo G. (2019). A carbon-supported BiSn nanoparticles based novel sensor for sensitive electrochemical determination of Cd (II) ions. Talanta.

[102] Ting S.L., Ee S.J., Ananthanarayanan A., Leong K.C., Chen P. (2015). Graphene quantum dots functionalized gold nanoparticles for sensitive electrochemical detection of heavy metal ions. Electrochim. Acta.

[103] Yang D., Wang L., Chen Z., Megharaj M., Naidu R. (2014). Voltammetric Determination of Lead (II) and Cadmium (II) Using a Bismuth Film Electrode Modified with Mesoporous Silica Nanoparticles. Electrochim. Acta.

[104] Zhou M., Han L., Deng D., Zhang Z., He H., Zhang L., Luo L. (2019). 4-mercaptobenzoic acid modified silver nanoparticles-enhanced electrochemical sensor for highly sensitive detection of Cu^2+^. Sens. Actuators B Chem..

[105] Zhu Y., Pan D., Hu X., Han H., Lin M., Wang C. (2017). An electrochemical sensor based on reduced graphene oxide/gold nanoparticles modified electrode for determination of iron in coastal waters. Sens. Actuators B Chem..

[106] Cheng Y., Fa H., Yin W., Hou C., Huo D., Liu F., Zhang Y., Chen C. (2016). A sensitive electrochemical sensor for lead based on gold nanoparticles/nitrogen-doped graphene composites functionalized with l-cysteine-modified electrode. J. Solid State Electrochem..

[107] Farzin L., Shamsipur M., Sheibani S. (2017). A review: Aptamer-based analytical strategies using the nanomaterials for environmental and human monitoring of toxic heavy metals. Talanta.

[108] Çiftçi H., Tamer U., Metin A.Ü., Alver E., Kizir N. (2014). Electrochemical copper (II) sensor based on chitosan covered gold nanoparticles. J. Appl. Electrochem..

[109] Zhang X.-F., Liu Z.-G., Shen W., Gurunathan S. (2016). Silver Nanoparticles: Synthesis, Characterization, Properties, Applications, and Therapeutic Approaches. Int. J. Mol. Sci..

[110] Renedo O.D., Martínez M.J.A. (2007). A novel method for the anodic stripping voltammetry determination of Sb (III) using silver nanoparticle-modified screen-printed electrodes. Electrochem. Commun..

[111] Han T., Jin J., Wang C., Sun Y., Zhang Y., Liu Y. (2017). Ag Nanoparticles-Modified 3D Graphene Foam for Binder-Free Electrodes of Electrochemical Sensors. Nanomaterials.

[112] Wu T., Xu T., Ma Z. (2015). Sensitive electrochemical detection of copper ions based on the copper(ii) ion assisted etching of Au@Ag nanoparticles. Analyst.

[113] Buledi J.A., Amin S., Haider S.I., Bhanger M.I., Solangi A.R. (2020). A review on detection of heavy metals from aqueous media using nanomaterial-based sensors. Environ. Sci. Pollut. Res..

[114] Cui L., Wu J., Li J., Ge Y., Ju H. (2014). Electrochemical detection of Cu^2+^ through Ag nanoparticle assembly regulated by copper-catalyzed oxidation of cysteamine. Biosens. Bioelectron..

[115] Mahmoudian M.R., Basirun W.J., Woi P.M., Yousefi R., Alias Y. (2019). l-Glutamine-assisted synthesis of ZnO oatmeal-like/silver composites as an electrochemical sensor for Pb^2+^ detection. Anal. Bioanal. Chem..

[116] Zhao Y., Zhang Z., Dang H. (2004). A simple way to prepare bismuth nanoparticles. Mater. Lett..

[117] He Y., Ma L., Zhou L., Liu G., Jiang Y., Gao J. (2020). Preparation and Application of Bismuth/MXene Nano-Composite as Electrochemical Sensor for Heavy Metal Ions Detection. Nanomaterials.

[118] Jeelani P.G., Mulay P., Venkat R., Ramalingam C. (2020). Multifaceted Application of Silica Nanoparticles. A Review. Silicon.

[119] Bapat G., Labade C., Chaudhari A., Zinjarde S. (2016). Silica nanoparticle based techniques for extraction, detection, and degradation of pesticides. Adv. Colloid Interface Sci..

[120] Fang Y., Cui B., Huang J., Wang L. (2019). Ultrasensitive electrochemical sensor for simultaneous determination of cadmium and lead ions based on one-step co-electropolymerization strategy. Sens. Actuators B Chem..

[121] Ul Hassan Alvi N., Gómez V.J., Soto Rodriguez P.E.D., Kumar P., Zaman S., Willander M., Nötzel R. (2013). An InN/InGaN quantum dot electrochemical biosensor for clinical diagnosis. Sensors.

[122] Bhanjana G., Dilbaghi N., Kumar R., Kumar S. (2015). Zinc Oxide Quantum Dots as Efficient Electron Mediator for Ultrasensitive and Selective Electrochemical Sensing of Mercury. Electrochim. Acta.

[123] Li L., Liu D., Shi A., You T. (2018). Simultaneous stripping determination of cadmium and lead ions based on the N-doped carbon quantum dots-graphene oxide hybrid. Sens. Actuators B Chem..

[124] Bakhsh E.M., Khan S.B., Marwani H.M., Danish E.Y., Asiri A.M. (2019). Efficient electrochemical detection and extraction of copper ions using ZnSe–CdSe/SiO_2_ core–shell nanomaterial. J. Ind. Eng. Chem..

[125] Hwang J.-H., Pathak P., Wang X., Rodriguez K.L., Park J., Cho H.J., Lee W.H. (2019). A novel Fe-Chitosan-coated carbon electrode sensor for in situ As (III) detection in mining wastewater and soil leachate. Sens. Actuators B Chem..

[126] Ma L., Zhang X., Ikram M., Ullah M., Wu H., Shi K. (2020). Controllable synthesis of an intercalated ZIF-67/EG structure for the detection of ultratrace Cd^2+^, Cu^2+^, Hg^2+^ and Pb^2+^ ions. Chem. Eng. J..

[127] Hu R., Zhang X., Chi K.-N., Yang T., Yang Y. (2020). Bifunctional MOFs-Based Ratiometric Electrochemical Sensor for Multiplex Heavy Metal Ions. ACS Appl. Mater. Interfaces.

[128] Lee S., Oh J., Kim D., Piao Y. (2016). A sensitive electrochemical sensor using an iron oxide/graphene composite for the simultaneous detection of heavy metal ions. Talanta.

[129] Anu Prathap M.U., Kaur B., Srivastava R. (2019). Electrochemical Sensor Platforms Based on Nanostructured Metal Oxides, and Zeolite-Based Materials. Chem. Rec..

[130] Yaqoob A.A., Parveen T., Umar K., Mohamad Ibrahim M.N. (2020). Role of Nanomaterials in the Treatment of Wastewater: A Review. Water.

[131] Shen Y.F., Tang J., Nie Z.H., Wang Y.D., Ren Y., Zuo L. (2009). Preparation and application of magnetic Fe_3_O_4_ nanoparticles for wastewater purification. Sep. Purif. Technol..

[132] Ali A., Hira Zafar M.Z., ul Haq I., Phull A.R., Ali J.S., Hussain A. (2016). Synthesis, characterization, applications, and challenges of iron oxide nanoparticles. Nanotechnol. Sci. Appl..

[133] Blaney L. (2007). Magnetite (Fe_3_O_4_): Properties, Synthesis, and Applications. http://preserve.lehigh.edu/cas-lehighreview-vol-15/5.

[134] Wu W., Jia M., Zhang Z., Chen X., Zhang Q., Zhang W., Li P., Chen L. (2019). Sensitive, selective and simultaneous electrochemical detection of multiple heavy metals in environment and food using a lowcost Fe_3_O_4_ nanoparticles/fluorinated multi-walled carbon nanotubes sensor. Ecotoxicol. Environ. Saf..

[135] Li S.-S., Zhou W.-Y., Jiang M., Guo Z., Liu J.-H., Zhang L., Huang X.-J. (2018). Surface Fe(II)/Fe(III) cycle promoted ultra-highly sensitive electrochemical sensing of arsenic (III) with dumbbell-like Au/Fe_3_O_4_ nanoparticles. Anal. Chem..

[136] Benício L.P.F., Silva R.A., Lopes J.A., Eulálio D., Dos Santos R.M.M., De Aquino L.A., Vergütz L., Novais R.F., Da Costa L.M., Pinto F.G. (2015). Layered double hydroxides: Nanomaterials for applications in agriculture. Rev. Bras. Cienc. Solo.

[137] Nalawade P., Aware B., Kadam V.J., Hirlekar R.S. (2009). Layered double hydroxides: A review. J. Sci. Ind. Res..

[138] Wang Q., O’Hare D. (2012). Recent Advances in the Synthesis and Application of Layered Double Hydroxide (LDH) Nanosheets. Chem. Rev..

[139] Forticaux A., Dang L., Liang H., Jin S. (2015). Controlled Synthesis of Layered Double Hydroxide Nanoplates Driven by Screw Dislocations. Nano Lett..

[140] Munonde T.S., Zheng H., Nomngongo P.N. (2019). Ultrasonic exfoliation of NiFe LDH/CB nanosheets for enhanced oxygen evolution catalysis. Ultrason. Sonochemistry.

[141] Munonde T.S., Zheng H., Matseke M.S., Nomngongo P.N., Wang Y., Tsiakaras P. (2020). A green approach for enhancing the electrocatalytic activity and stability of NiFe_2_O_4_/CB nanospheres towards hydrogen production. Renew. Energy.

[142] Asadpour-Zeynali K., Amini R. (2017). A novel voltammetric sensor for mercury(II) based on mercaptocarboxylic acid intercalated layered double hydroxide nanoparticles modified electrode. Sens. Actuators B Chem..

[143] Xu Z., Fan X., Ma Q., Tang B., Lu Z., Zhang J., Mo G., Ye J., Ye J. (2019). A sensitive electrochemical sensor for simultaneous voltammetric sensing of cadmium and lead based on Fe_3_O_4_/multiwalled carbon nanotube/laser scribed graphene composites functionalized with chitosan modified electrode. Mater. Chem. Phys..

[144] Singh S., Pankaj A., Mishra S., Tewari K., Singh S.P. (2019). Cerium oxide-catalyzed chemical vapor deposition grown carbon nanofibers for electrochemical detection of Pb(II) and Cu(II). J. Environ. Chem. Eng..

[145] Salimi A., Pourbahram B., Mansouri-Majd S., Hallaj R. (2015). Manganese oxide nanoflakes/multi-walled carbon nanotubes/chitosan nanocomposite modified glassy carbon electrode as a novel electrochemical sensor for chromium (III) detection. Electrochim. Acta.

[146] Zhang Z., Ji H., Song Y., Zhang S., Wang M., Jia C., Tian J.-Y., He L., Zhang X., Liu C.-S. (2017). Fe (III)-based metal–organic framework-derived core–shell nanostructure: Sensitive electrochemical platform for high trace determination of heavy metal ions. Biosens. Bioelectron..

[147] Oliveira V.H.B., Rechotnek F., da Silva E.P., de Sousa Marques V., Rubira A.F., Silva R., Lourenço S.A., Muniz E.C. (2020). A sensitive electrochemical sensor for Pb^2+^ ions based on ZnO nanofibers functionalized by L-cysteine. J. Mol. Liq..

[148] Yu L., Zhang P., Dai H., Chen L., Ma H., Lin M., Shen D. (2017). An electrochemical sensor based on Co_3_O_4_ nanosheets for lead ions determination. RSC Adv..

[149] Peixoto A.C., Silva A.F. (2017). Smart devices: Micro-and nanosensors. Bioinspired Materials for Medical Applications.

[150] Hunter G.W., Stetter J.R., Hesketh P., Liu C.-C. (2010). Smart Sensor Systems. Electrochem. Soc. Interface.

[151] Kumar R. (2018). Smart Micro/Nano Sensors and their Applications in Intelligent Sensory Network System. Int. J. Sens. Netw. Data Commun..

[152] Dong B., Shi Q., Yang Y., Wen F., Zhang Z., Lee C. (2020). Technology evolution from self-powered sensors to AIoT enabled smart homes. Nano Energy.

[153] Saini R.K., Bagri L.P., Bajpai A.K. (2017). Smart nanosensors for pesticide detection. New Pesticides and Soil Sensors.

